# Serum Peptidome Variations in a Healthy Population: Reference to Identify Cancer-Specific Peptides

**DOI:** 10.1371/journal.pone.0063724

**Published:** 2013-05-08

**Authors:** Kun He, Xin-Yu Wen, Ai-Ling Li, Tao Li, Jie Wang, Hong-Xia Wang, Na Wang

**Affiliations:** 1 Institute of Basic Medical Sciences, National Center of Biomedical Analysis, Beijing, China; 2 The 301 General Hospital, Beijing, China; I2MC INSERM UMR U1048, France

## Abstract

The emergence of mass spectrometry (MS)-based signatures as biomarkers has generated considerable enthusiasm among oncologists. However, variations in normal individuals also exist, and a better understanding of serum peptide patterns of healthy individuals will be important for further exploring disease-specific serum peptide patterns. Following development of a serum peptide pattern platform, we analyzed 500 serum samples obtained from healthy individuals. Samples from breast (n = 84), lung (n = 70), and rectal (n = 30) cancer patients were also examined. Extensive data analysis revealed negligible contributions of age to serum peptide patterns except in healthy individuals between 20–30 and 60+ years of age. Gender-related variations in the serum patterns of healthy individuals were only observed in 20–30 year-old individuals. Our results revealed substantial variation in individual peptide profiles, but 65 peptides were detected at a 20% higher frequency in the healthy population. A peptide profile was developed for each type of cancer, containing 10 discriminating peptides not prevalent in healthy individuals. Sequence identification of 111 signature peptides revealed that they fell into several tight clusters and most were exopeptidase products of serum resident proteins. We have obtained a MS-based serum peptide profile for healthy individuals, providing a reference for observing the occurrence of cancer-specific peptides.

## Introduction

The emergence of mass spectrometry (MS)-based signatures as biomarkers has generated considerable enthusiasm among oncologists and analytical chemists [1]. MS techniques permit the resolution of hundreds of small- to medium-sized peptides from only microliters of serum. Villanueva and colleagues demonstrated that cancer can be detected and classified based on serum peptides generated as a result of tumor protease activity [2]. These so-called proteomic patterns entered mainstream proteomics with the publication of a Lancet study in 2002, which was quickly followed by a significant number of research studies and reviews. Several recent reports have highlighted MS-based determinations of specific peptide patterns that indicate the presence of tumors with very high sensitivity and specificity [3]–[16]. These studies have been performed on high resolution instruments using matrix-assisted laser desorption/ionization (MALDI) ion sources and time-of-flight (TOF) ion detectors [2], [10]–[16]. More sophisticated instrumentation such as Fourier Transform (FT) MS/MS permits identification of a larger number of peptide sequences^15^. Recently, affinity bead–based purification has been developed and adopted by some investigators, since spherical beads have larger combined surface areas and a higher binding capacity than small-diameter spots [14]–[19].

The pattern of serum peptides produced by various disease states holds a great deal of disease-specific diagnostic information. It is hoped that panels of tens to hundreds of peptide markers can transcend the heterogeneity of serum samples to generate a high level of diagnostic specificity. Measuring panels of peptidome markers might be more sensitive and specific than conventional biomarker approaches. However, critics have voiced concerns regarding several sources of bias in serum proteomics-based biomarker discovery. Among them, age and gender are the main sources of bias that may affect the composition and relative abundance of serum peptides [20]. Therefore, a better understanding of peptide patterns in serum from healthy individuals will be important for further exploring disease-specific serum peptide profiles.

In this study, we address whether the parameters of gender and age influence serum peptide patterns in healthy individuals. The study included 500 serum samples from healthy men and women between 20 and 80 years of age. Samples from breast, lung, and rectal cancer patients were examined as well. Extensive data analysis revealed negligible contributions of age to serum peptide patterns except in healthy individuals between 20–30 and 60+ years of age. and that gender-related variation in serum patterns in healthy individuals occurred only between 20–30 years of age. Our results showed that peptide profiles varied individually, but that 65 peptides were detected at a 20% higher frequency in the healthy population. A peptide profile was developed for each type of cancer, containing 10 discriminating peptides not prevalent in healthy individuals. Sequence identification of 111 signature peptides revealed that they fell into several tight clusters and most were exopeptidase products of serum resident proteins

## Materials and Methods

### Reagent

Purification kit copper-chelated magnetic bead was prepared from our lab[21]. Calibration standards containing nine peptides were used as artificial markers (Bruker Daltonik) and consisted of the following molecules with average molecular masses given in parentheses: angiotensin II (1046.54180), angiotensin I (1296.68480), substance P (1347.73540), bombesin (1619.82230), ACTH clip1-17 (2093.08620), ACTH clip18–39 (2465.19830), somatostatin (3147.47100), Ins bov(5734.55700), ubiquitin(8565.88500).

### The ethics statement

After we obtained approval from the ethics committee of 301 General Hospital and the ethics committee of the first clinical hospital of Jilin university, all donors had signed the consent forms.

### Serum samples

Blood samples from healthy volunteers and were collected from 301 General Hospital and subjected to physical examination and defined as clinical healthy. Patients diagnosed with lung cancer, breast cancer and rectal cancer were collected from the first clinical hospital of Jilin university (Table S1, Table S2 and Table S3). All samples were collected following standard clinical protocol. The no-anticoagulated blood samples were collected in the morning before the donors had breakfast and allowed to clot at room temperature for two hours, and centrifuged at 1500–2000 rpm for 15 min. Sera were transferred to four cryovials, and stored frozen at −80°C until further use. For the reproducibility experiments, sera from blood samples of 10 healthy volunteers were pooled as quality control sera. When serum arrived the mass spectrometry (MS) lab, each cryovial of sample was thawed on ice and used to generate ten smaller aliquots (20 µL each) in micro-eppendorf tubes and stored at −80°C until further use. Each serum sample had therefore been frozen and thawed twice before it was subjected to peptide extraction and MS analysis.

### Copper-chelated magnetic beads peptide extraction and MALDI-TOF-MS (Mass Spectrometry)

Copper-chelated magnetic beads prepared in our lab were used for extracting peptides of serum. A 5-µL aliquot of a homogeneous magnetic beads solution was transferred to a 0.2-mL thin-walled PCR tube, and 50 µL of binding solution was added and mixed thoroughly. The tubes were placed in a magnetic bead separator (MBS) to fix the magnetic beads, and the supernatant was aspirated. After the binding step, 20 µL binding solution was added and mixed in the PCR-tube. Then, 5 µL serum sample was added, mixed and then placed in the MBS device. The supernatant was aspirated, and 100 µL of wash solution was added and mixed with the magnetic beads. After final washing step, bound peptides were eluted by incubation of 20 µL elution solution for 10 min before collecting the elution solution using the MBS device. 1 µL of the eluent was mixed with 1 µL of matrix (HCCA) solution. 1 µL was spotted onto a 600 µm-diameter spot size 384 MTP target plate (Bruker Daltonik) and left to dry. The peptide and protein calibration standard (1 µL) in the same matrix was applied to target spots in proximity to the serum samples for external calibration of the instrument. The processed samples were analyzed by a liner MALDI-TOF/TOF mass spectrometer (Ultraflex; Bruker Daltonics) equipped with a pulsed ion extraction source. The MALDI-TOF-MS system was controlled by flex control software v.2.0 (Bruker Daltonics). Ionization was achieved by irradiation with a nitrogen laser (337 nm) operating at 4 Hz. Ions were accelerated at 20 kV with 250 ns of pulsed ion extraction delay. Each spectrum was detected in linear positive mode and was externally calibrated using a mixture of peptide/protein standards between 1000 and 10000 Da.

### Peptide sequencing

FT-ICR tandem mass spectrometer (Bruker Daltonics) is equipped with a 9.4 T superconducting magnet and nano HPLC system. We used nanoESI source equipped with an angled off-axis spraying system in the positive ion mode. The voltage applied to the metal-coated capillary entrance of the electrospray source was set between-1200 V and −1600 V. The extracted peptides were desalted and sequenced by autoMSn mode with MS/MS boost function. The Q FTMS was set up to do MS/MS scan. The selected ion was fragmented by collision-induced dissociation (CID). All mass spectra were acquired in the broadband mode in the mass range from m/z 300 to 3000 with 512 k data points. The spectra were calibrated externally with Agilent ES tuning mix (catalog number G2421A, Palo Alto, CA) by four-point calibration. The instrument was controlled using software Apex 1.0 and the data was analyzed by DataAnalysis 3.4. (Bruker Daltonics). All MS/MS data in a run were deconvoluted and then transferred to a file (.mgf), and were input into the search engine Mascot (www.matrixscience.com). Peptide mass tolerance was set at 10 ppm, fragment ion mass tolerance was set at 0.01, and the mass type of parent peptide and peptide fragment were set at monoisotopic.

### Signal processing

Spectra were converted into excel file format included m/z value and signal intensities using the FlexAnalysis 2.0 software. Every single spectrum was smoothed curve, baseline subtraction and peak labeling. All spectra qualified mass peaks (signal-to-noise ratio>5) with mass-to-charge ratios (m/z) between 1000 and 10000 Dalton were compiled labeled. All peak intensities were normalized to the total ion current of m/z and all spectra were then taken for alignment. We normalized and aligned peaks from distinct spectra using our MatchPeaks software. Alignment starts with the first peak [whose mass to-charge ratio (m/z) is denoted by X] as an “anchor”. If another peak has m/z  =  Y, we define the relative mass error to be |X _ Y|/X. The software identifies two peaks if the relative mass error is >0.3%. After normalization and alignment, peak lists were then binned by using the resolution of the peaks and a spreadsheet was created for further statistical analysis.

### Classification

SIMCA P+™ software, developed in Umetrics, was also used for visualization and statistical analysis different age groups of 500 healthy individuals. After signal processing, a graphical viewer for spectra in excel format. The SIMCA P+ software begin to analyze data using statistical algorithm, Principle Component Analysis (PCA), Partial Least Square (PLS) and Orthoganal Signal Correction (OSC) and then export Mass Spectra Viewer, a visual interface for processed spectral data, plots. Finally in this paper, we analyzed all data using a straightforward chemometric approach called Partial Least Squares- Discriminant Analysis (PLS-DA)

## Results

### Serum peptide profiling

**Figure 1:**
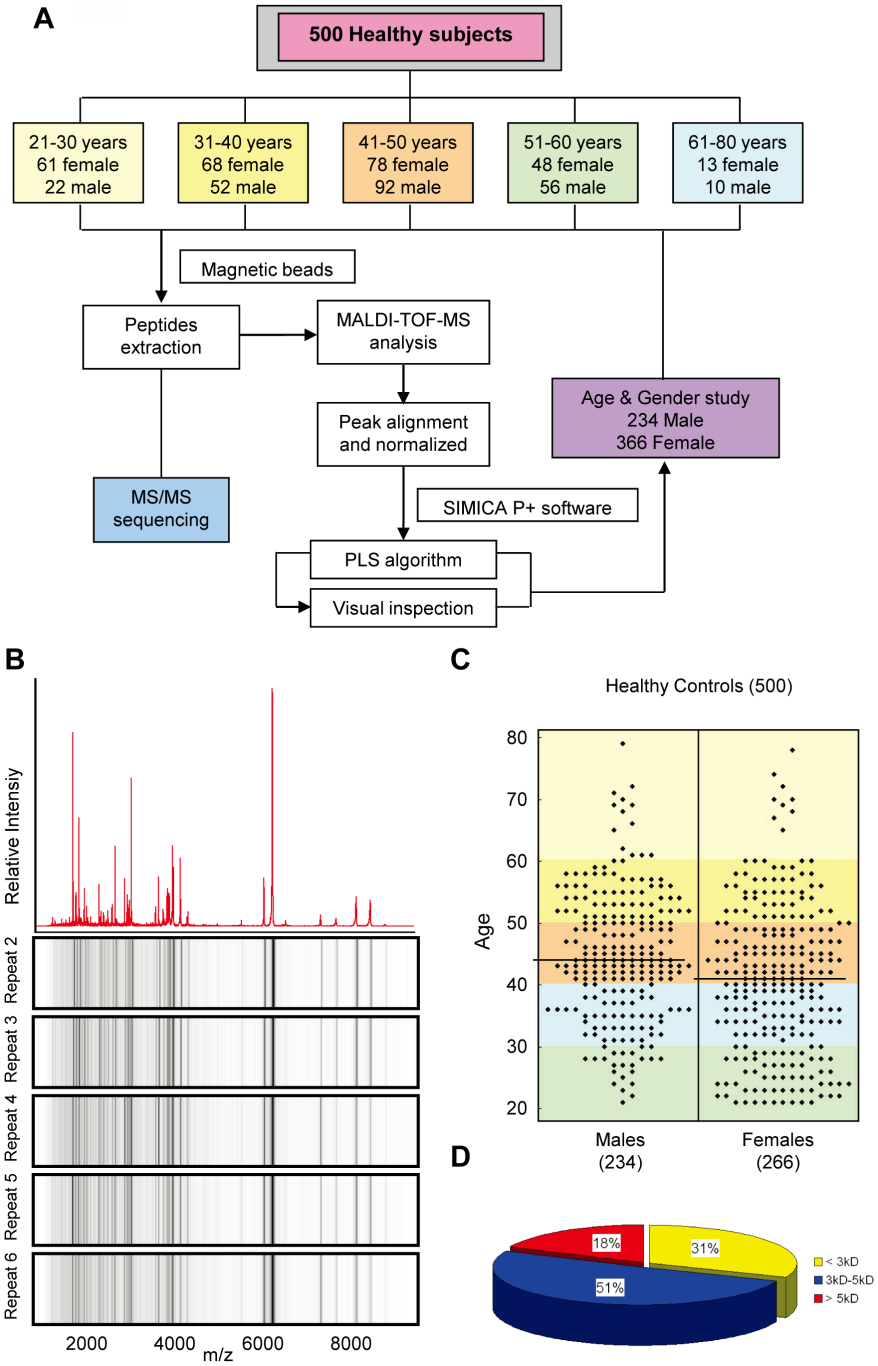
Peptide profiles in healthy subjects. (**A**) Study overview. The diagram shows the design of the healthy serum peptidome demographic study, and the statistical analysis approach used for gender and different age groups. (**B**) Example of within-run reproducibility of mass spectra. Serum from a pooled healthy sample (10 individuals) was prepared with copper-chelated magnetic bead extraction. Six in one day randomly selected spectra obtained by MALDI-TOF MS and pseudo gel spectra used in the analysis. The analysis m/z range is from 1 KD to 10 KD. (**C**) Age distribution of 500 healthy subjects. The colored fields indicate the five age groups used throughout this study. The black horizontal lines represent medians for each cohort. (**D**) Distribution of 65 peptides with their molecular weight ranges.

The serum profile of copper-chelated magnetic bead-binding peptides was detected using MALDI-TOF MS (Figure 1A) and displayed according to mass:charge ratio (m/z) (Figure 1B). Sample handling after collection was uniform, involving 2 freeze-thaw cycles to store and aliquot samples for peptide extraction and MS analysis. Peptides were extracted on copper-chelated magnetic beads, and were washed, eluted, mixed with matrix, and deposited on the MALDI target plate, followed by MALDI-TOF MS analysis. The system reproducibility was first determined with respect to relative peak intensities (Figure 1B). Samples from 500 healthy individuals of various ages were randomly distributed during processing and analysis. After the processed spectra were aligned, the data for each sample was statistically analyzed. Peptide profiles for all samples were analyzed using the Partial Least-Squares (PLS) algorithm (Figure 1A) contained in the SIMCA P+ software package. The coefficient of variance (CV) for peak intensity was calculated using 10 randomly chosen peaks with a signal:noise ratio >5 and m/z 1–10 kDa representing low-, medium-, and high-molecular weight peptides. Maximum mass shifts over the range 1–10 kDa were 0.012% for the peptide calibration mixture and 0.018% for the pooled serum sample. These results correlate with a variation of <1Da in the absolute mass shift for signals <3000 Da. The within-day (Figure 1B, Table S4) and between-day (Table S4) CVs for direct MS analysis of the peptides were both <15%.

### Serum peptidome variability

**Figure 2:**
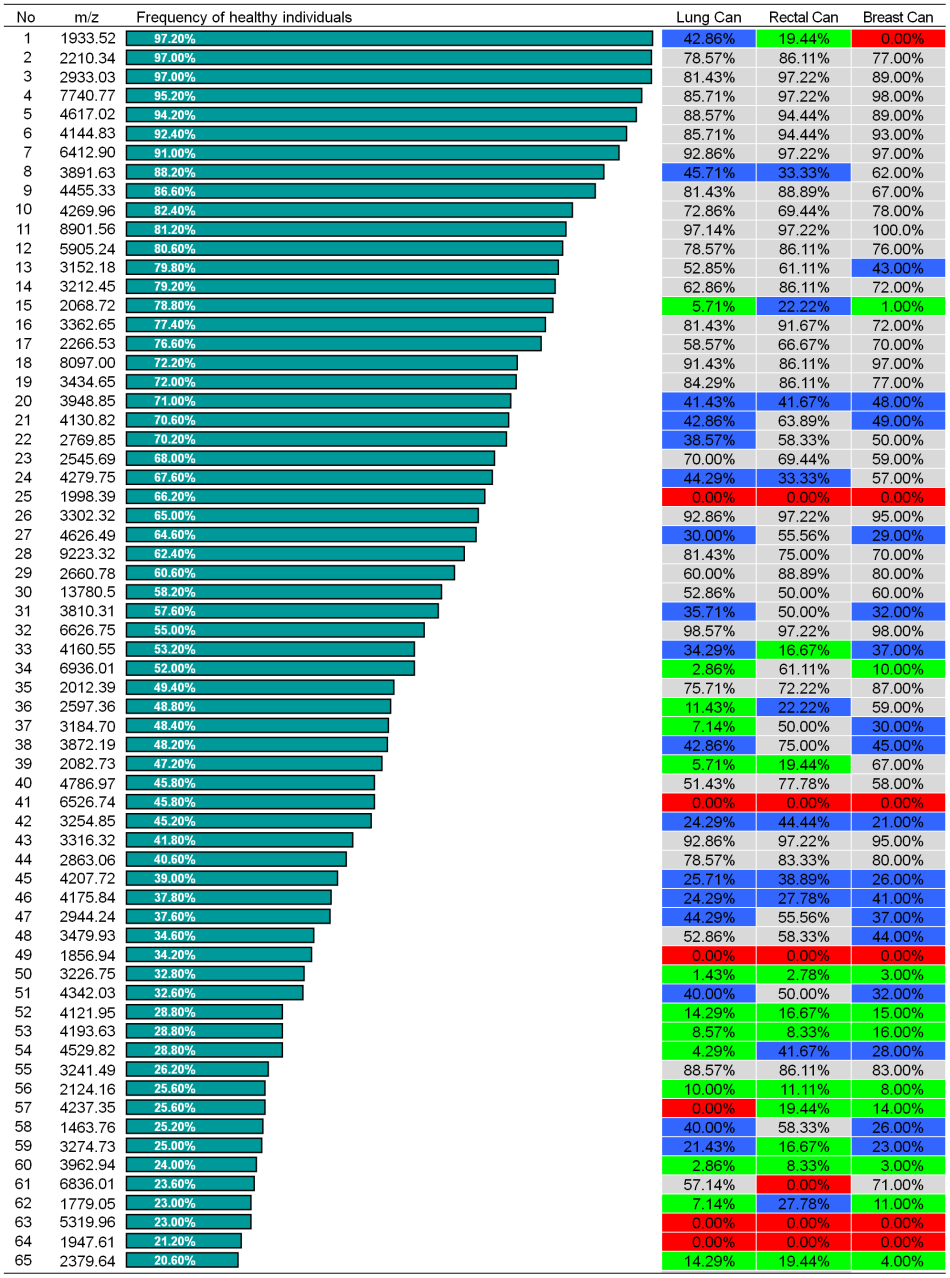
Occurrence frequency of 65 peptides in the sera of healthy individuals and patients with three types of cancer. m/z (mass:charge ratio) indicates protonated average molecule weight (M+H) in linear MALDI-TOF-MS. Red: zero frequency in cancers; Green: <20% frequency; Blue: 20–50% frequency; Grey: >50%.

To evaluate serum peptidome diversity and the possible effects of major demographic parameters such as gender and age on serum peptide profiling, we collected blood from 500 healthy volunteers and prepared the samples using a standard clinical protocol. Volunteers were selected for the study to yield five age groups: 20–30 years old (61 women and 22 men), 30–40 years old (68 women and 52 men), 40–50 years old (78 women and 92 men), 50–60 years old (48 women and 56 men), and over 60 years old (13 women and 10 men) (Figure 1C). Serum peptide profiles with similar chelating motifs were affinity-retrieved simultaneously. The overall peptide profiles of all samples were analyzed. Though the peptide profiles varied individually, 65 peptides were detected in a frequency of more than 20% in the healthy population, and their m/z and frequency of occurrence are listed in Figure 2. The majority of the selected peaks corresponded to peptides with molecular mass less than 5,000 Da (Figure 1D).

**Figure 3:**
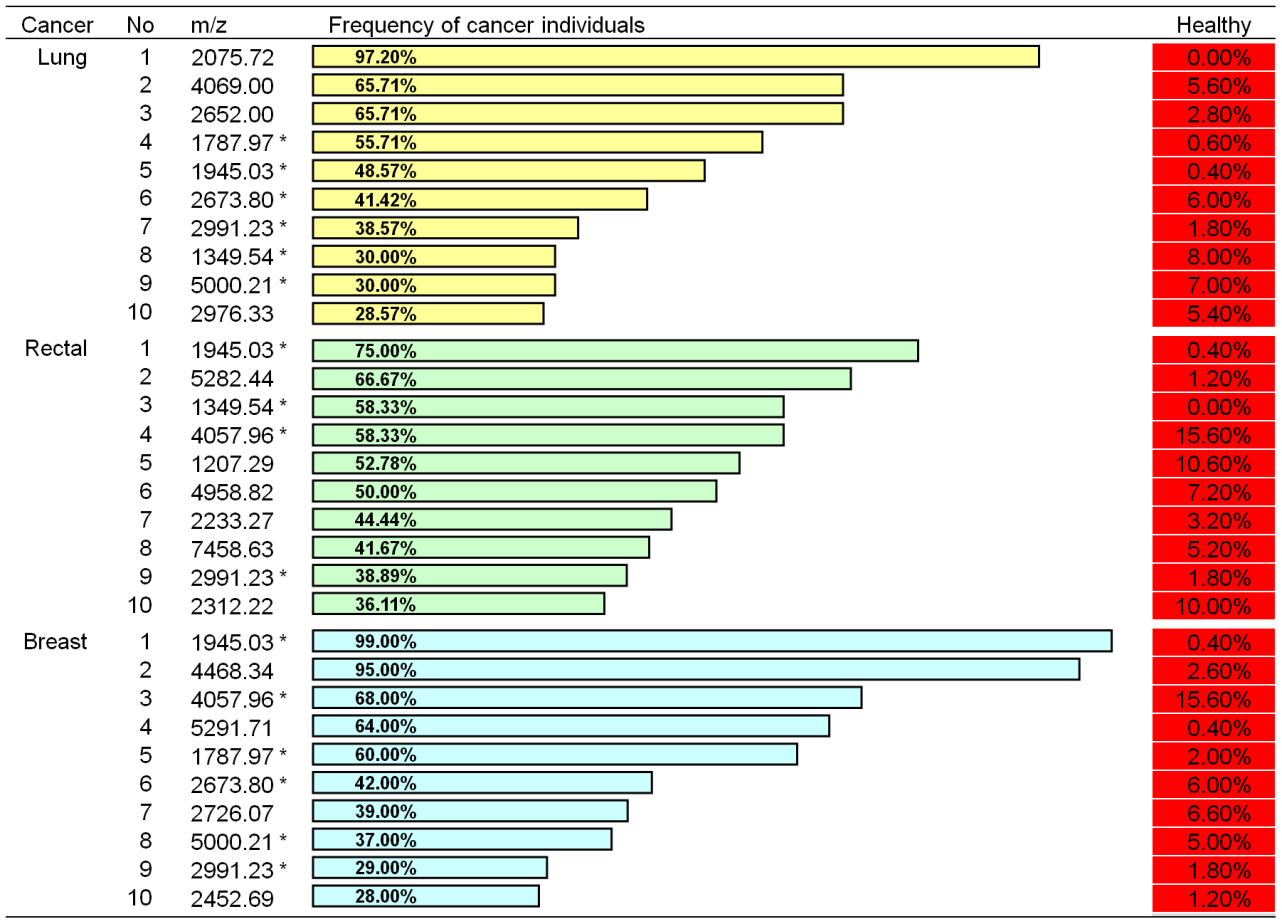
Peptides with >20% frequency in the sera of cancer patients and <20% frequency in healthy individuals. Red asterisk: occurrence in three cancers with >20% frequency; Blue asterisk: occurrence in two of the three cancers with >20% frequency.

To investigate whether the 65 peptides represented the serum peptide pattern of a healthy population, we also analyzed the occurrence frequency of the 65 peptides in sera of breast (n = 84), lung (n = 70), and rectal (n = 30) cancer patients. Our results showed that the majority of the 65 peptides were detected in the cancer patients with a frequency that was significantly different from the healthy population (Figure 2). It suggests that the 65 peptides can be developed into a signature for healthy individuals and provide a reference for defining cancer-specific peptide patterns. On the other hand, we discovered 3 sets of 10 distinctive peptides with a high frequency in the three different cancers respectively (Figure 3), and found that they had not been observed in the healthy population (Figure 2).

**Figure 4:**
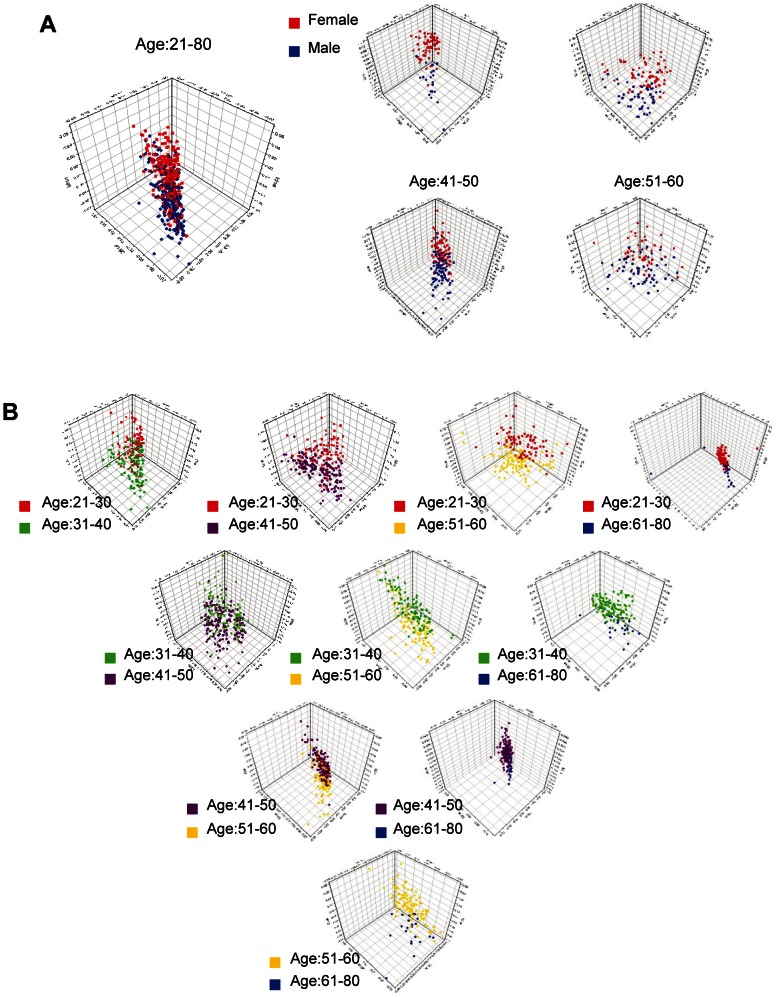
PLS analysis on the age and gender-related variations of the serum peptide profiles of healthy individuals. (**A**) 3D scatter plots of PLS analysis on the serum peptide profiles derived from male (blue) and female (red) donors with different age ranges. **(B)** 3D scatter plots of PLS analysis on age-related variation of serum peptide profiles.

The gender-associated serum peptide variability was negligible in the 500 samples (Figure 4A), except in the group of 20–30 year-olds. In the 20–30 age group, the first five PLS components had an R^2^ value of 0.82. With increasing age, the gender variability became lower, and the first five PLS components had R^2^ values less than 0.5. Our study also showed negligible age-associated variability in comparisons between most groups. Figure 4B contains 3D scatter plots of the first three PLS components of every pair of age groups. A significant difference appeared only in the pairing between the 20–30 and 60–80 year-old groups, where the first five PLS components had an R^2^ value of 0.64. Overall, the closer two age groups were, the less the variability (Figure 4B).

### Serum peptide identification

**Figure 5:**
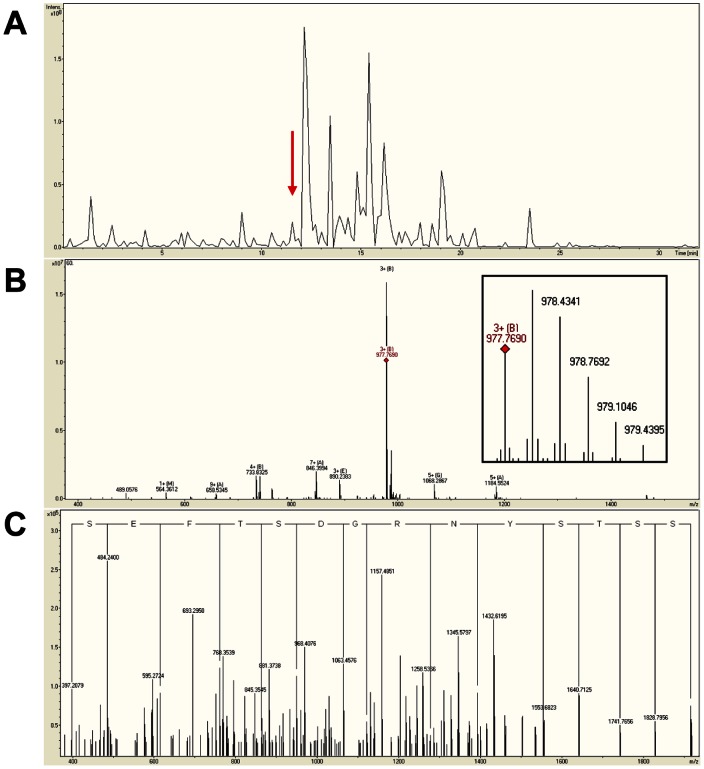
NanoLC-FT-ICR MS/MS identification of a peptide, which is a fragment of protein Fibrinogen α, with sequence SSSYSKQFTSSTSYNRGDSTFESKSY and molecular weight 2930.2842 Da. (A) Base peak ion chromatogram of serum peptides. The arrow indicates the peak including the triple-charged peptide at m/z 977.7690 (B) Mass spectrum of the ion chromatography peak indicated in (A), the diamond indicates the precursor ion at m/z 977.7690. The insert is a magnified view of the precursor ion showing a triple-charged ion at high mass accuracy (0.3 ppm). (C) Tandem mass spectrum of the triple-charged peptide at m/z 977.7690 with amino acid sequence deduced from the y ion series. Note that y ions originate at the C terminus and the sequence therefore reads backwards.

To get higher confidence peptide identifications, we employed a hybrid FT-ICR MS/MS with a 9.4 T superconducting magnet and nano-HPLC system to acquire accurate mass measurements (within 2 ppm). Of the peptides from healthy individuals and the three groups of cancer patients, 111 were positively identified by database searches using MASCOT (Table 1). Note that the m/z values listed in Table 1 are monoisotopic and average isotopic values + H [M+H] (consistent with the results obtained with MALDI). Figure 5 depicts a typical example of the data obtained for the peptide SSSYSKQFTSSTSYNRGDSTFESKSY (Fibrinogen α), which was identified as a triple-charged ion with mass accuracy of 0.3 ppm. The 111 peptides were derived from 20 naturally occurring serum proteins, including fibrinopeptide α (FPA), ITIH4 and C3f. Interestingly, all but a few peptide sequences clustered into sets of overlapping fragments aligned within each group at either the C or N terminus and with ladder-like truncations at the opposite ends. The sequence assignments either had above-threshold scores or were unequivocally assigned as the precursor ion mass, and selected fragment ion masses (y or b) matched a particular rung in the ladder.

**Table 1:** Identification of serum peptides.

Protein name	No.	M_mon_	M_avg_+H	Sequence
Fibrinogen a	1	1535.69	1537.58	ADSGEGDFLAEGGGVR
	2	1464.65	1466.50	DSGEGDFLAEGGGVR
	3	1349.62	1351.42	SGEGDFLAEGGGVR
	4	1262.59	1264.34	GEGDFLAEGGGVR
	5	1205.57	1207.29	EGDFLAEGGGVR
	6	1076.53	1078.17	GDFLAEGGGVR
	7	1019.50	1021.12	DFLAEGGGVR
	8	1308.55	1310.32	DSGEGDFLAEGGGV
	9	1193.52	1195.23	SGEGDFLAEGGGV
	10	920.42	921.98	GDFLAEGGGV
	11	863.40	864.93	DFLAEGGGV
	12	748.38	749.84	FLAEGGGV
	13	649.31	650.71	FLAEGGG
	14	707.31	708.75	DFLAEGG
	15	1389.62	1391.44	NRGDSTFESKSY
	16	2552.09	2554.60	SSSYSKQFTSSTSYNRGDSTFES
	17	2767.22	2769.85	SSSYSKQFTSSTSYNRGDSTFESKS
	18	2930.28	2933.03	SSSYSKQFTSSTSYNRGDSTFESKSY
	19	3189.42	3192.40	SSSYSKQFTSSTSYNRGDSTFESKSYKM
	20	4783.09	4786.97	SSSYSKQFTSSTSYNRGDSTFESKSYKMADEAGSEADHGTHSTK
	21	4996.21	5000.21	SSSYSKQFTSSTSYNRGDSTFESKSYKMADEAGSEADHGTHSTKRG
	22	5333.35	5337.55	SSSYSKQFTSSTSYNRGDSTFESKSYKMADEAGSEADHEGTHSTKRGHA
	23	5900.70	5905.24	SSSYSKQFTSSTSYNRGDSTFESKSYKMADEAGSEADHEGTHSTKRGHAKSRPV
	24	3522.53	3525.67	GDSTFESKSYKMADEAGSEADHEGTHSTKRGHA
	25	3238.52	3241.49	SYKMADEAGSEADHEGTHSTKRGHAKSRPV
	26	2671.17	2673.80	SYKMADEAGSEADHEGTHSTKRGHA
	27	2249.95	2252.34	SYKMADEAGSEADHEGTHSTK
	28	2121.85	2124.16	SYKMADEAGSEADHEGTHST
	29	2988.42	2991.23	KMADEAGSEADHEGTHSTKRGHAKSRPV
	30	2874.34	2877.08	MADEAGSEADH_CH3_EGTHSTKRGHAKSRPV
	31	2860.33	2863.06	MADEAGSEADHEGTHSTKRGHAKSRPV
	32	2761.26	2763.93	MADEAGSEADHEGTHSTKRGHAKSRP
	33	2292.98	2295.37	MADEAGSEADHEGTHSTKRGHA
	34	2729.29	2731.86	ADEAGSEADHEGTHSTKRGHAKSRPV
	35	2161.94	2164.17	ADEAGSEADHEGTHSTKRGHA
	36	2658.25	2660.78	DEAGSEADHEGTHSTKRGHAKSRPV
	37	2559.18	2561.65	DEAGSEADHEGTHSTKRGHAKSRP
	38	2090.90	2093.09	DEAGSEADHEGTHSTKRGHA
	39	2019.86	2022.01	DEAGSEADHEGTHSTKRGH
	40	1882.80	1884.87	DEAGSEADHEGTHSTKRG
	41	2543.22	2545.69	EAGSEADHEGTHSTKRGHAKSRPV
	42	2343.14	2345.50	GSEADHEGTHSTKRGHAKSRPV
	43	2815.32	2817.99	GSESGIFTNTKESSSHHPGIAEFPSRG
C3f	44	2020.10	2022.32	SSKITHRIHWESASLLR
	45	1305.69	1307.50	SKITHRIHWE
	46	1776.96	1779.05	SKITHRIHWESASLL
	47	1689.93	1691.97	KITHRIHWESASLL
	48	1448.75	1450.64	THRIHWESASLL
	49	977.48	979.09	THRIHWE
	50	1347.70	1349.54	HRIHWESASLL
	51	1054.54	1056.21	IHWESASLL
	52	941.46	943.05	HWESASLL
	53	955.48	957.07	H_CH3_WESASLL
	54	828.38	829.89	HWESASL
C3 precursor	55	667.37	668.77	ASHLGLA
	56	1854.85	1856.94	SEETKENEGFTVTAEGK
C4a	57	1895.02	1897.15	RNGFKSHALQLNNRQI
	58	1497.78	1499.67	NGFKSHALQLNNR
	59	895.45	896.98	SHALQLNN
	60	3199.79	3202.72	GLEEELQFSLGSKINVKVGGNSKGTLKVLR
	61	2703.44	2706.07	GLEEELQFSLGSKINVKVGGNSKGTL
	62	2304.20	2306.58	GLEEELQFSLGSKINVKVGGNS
	63	2377.20	2379.64	DDPDAPLQPVTPLQLFEGRRN
ITIH4	64	3271.63	3274.73	MNFRPGVLSSRQLGLPGPPDVPDHAAYHPF
	65	2723.38	2726.07	PGVLSSRQLGLPGPPDVPDHAAYHPF
	66	2626.33	2628.95	GVLSSRQLGLPGPPDVPDHAAYHPF
	67	2357.16	2359.61	SSRQLGLPGPPDVPDHAAYHPF
	68	2183.09	2185.45	QLGLPGPPDVPDHAAYHPFR
	69	2026.99	2029.26	QLGLPGPPDVPDHAAYHPF
	70	1785.85	1787.97	GLPGPPDVPDHAAYHPF
	71	1170.57	1172.28	GLPGPPDVPDHA
	72	1099.53	1101.20	GLPGPPDVPDH
	73	1000.46	1002.07	PGPPDVPDHA
	74	903.41	904.96	GPPDVPDHA
	75	832.37	833.88	GPPDVPDH
	76	3155.62	3158.59	NVHSGSTFFKYYLQGAKIPKPEASFSPR
	77	1388.71	1390.60	GSEMVVAGKLQDR
apoA-I	78	3374.74	3377.81	AELQEGARQKLHELQEKLSPLGEEM_CH3_RDRA
	79	3181.73	3184.70	QGLLPVLESFKVSFLSALEEYTKKLNTQ
	80	2052.07	2054.31	ATEHLSTLSEKAKPALEDL
apoA-IV	81	2507.35	2509.83	ISASAEELRQRLAPLAEDVRGNL
	82	2754.36	2756.99	GNTEGLQKSLAELGGHLDQQVEEFR
Kininogen HMW	83	2208.05	2210.34	KHNLGHGHKHERDQGHGHQ
	84	1942.90	1945.03	NLGHGHKHERDQGHGHQ
	85	2126.01	2128.28	GHGLGHGHEQQHGLGHGHKF
Transthyretin	86	2450.20	2452.69	ALGISPFHEHAEVVFTANDSGPR
	87	3156.61	3159.52	DSGPRRYTIAALLSPYSYSTTAVVTNPKE
	88	2040.04	2042.29	ALLSPYSYSTTAVVTNPKE
	89	1395.69	1397.53	SYSTTAVVTNPKE
	90	1409.70	1411.55	SYSTTAVVTNPK_CH3_E
Antichymotrypsin	91	4622.49	4626.49	SALVETRTIVRFNRPFLMIIVPTDTQNIFFMSKVTNPKQA
	92	4464.42	4468.34	LVETRTIVRFNRPFLMIIVPTDTQNIFFMSKVTNPKQA
	93	4351.33	4355.18	VETRTIVRFNRPFLMIIVPTDTQNIFFMSKVTNPKQA
	94	2735.44	2738.29	LMIIVPTDTQNIFFMSKVTNPKQA
	95	2491.31	2493.93	IIVPTDTQNIFFMSKVTNPKQA
α-1 antitrypsin	96	4772.55	4776.77	LEAIPMSIPPEVKFNKPFVFLMIDQNTKSPLFMGKVVNPTQK
	97	4118.21	4121.95	SIPPEVKFNKPFVFLMIDQNTKSPLFMGKVVNPTQK
	98	2488.32	2491.00	LMIDQNTKSPLFMGKVVNPTQK
proapolipoprotein	99	1365.71	1367.54	LEEYTKKLNTQ
Cytokeratin	100	1811.85	1813.88	SRSGGGGGGGLGSGGSIRSSY
Protein c inhibitor	101	3888.15	3891.63	SARLNSQRLVFNRPFLMFIVDNNILFLGKVNRP
	102	2195.18	2197.62	SARLNSQRLVFNRPFLMF
	103	2048.11	2050.44	SARLNSQRLVFNRPFLM
prealbumin	104	1437.83	1439.70	VKVLDAVRGSPAIN
	105	785.43	786.90	VVTNPKE
serum albumin	106	1295.64	1297.42	DAHKSEVAHRF
	107	1538.76	1540.68	DAHKSEVAHRFKD
SAA	108	1524.78	1526.74	PNHFRPAGLPEKY
SP40	109	4266.29	4269.96	PITVTVPVEVSRKNPKFMETVAEKALQEYRKKHREE
apolipoprotein CI	110	1461.86	1463.76	FQKVKEKLKIDS
C1-inhibitor	111	1063.52	1065.24	MGRVYDPRA

M_mon_ is the monoisotopic molecular weight. M_avg_+H is protonated average molecule weight as listed under m/z in Figure 2 and 3.

## Discussion

Serum peptidome diversity contains a rich variety of disease-specific diagnostic information. The composition of the serum peptidome is a true mirror of ongoing cellular and organ system function. Although this means that subsets of the serum peptidome can potentially reflect subtle disease events in small tissue volumes, it also means that the peptidome is constantly fluctuating in response to ongoing daily physiological events [1], [22]. Oncologists and clinical chemists fear that the level of individual peptides can be greatly influenced by various non-disease-related epidemiological factors and normal physiological conditions. Variations in normal individuals also exist and likely contribute to the difficulty in identifying disease-specific signals in serum peptide profiles. Thus, as with any biomarker discovery, great care is needed to reduce bias. In doing so, a large number of healthy individuals must be measured to define a healthy serum peptide profile. We have developed a unique platform to study a large cohort of identically collected and processed samples from 500 healthy men and women aged 20 to 80 years. Extensive analysis of MALDI-TOF MS data has suggested a negligible gender contribution to serum peptide patterns in all but those 20–30 years old. Our results further showed that there was a significant difference in peptide composition only in the pairing between the 20–30 and 60–80 year-old age groups. Moreover, the variability decreased as groups became closer in age.

Age and gender are the principal potential sources of bias that may affect the molecular patterns being evaluated for diagnostic and prognostic utility. Serum peptide profiles are affected by variations in gene expression and post-transcriptional events. Recently, Villanueva reported negligible differences in serum patterns among different age groups, but divided his data into age ranges of 20–35, 35–50 and 50–80 years (20). In our experiments, age groups were divided into 10-year cohorts except above age 60, and the comparisons produced consistent results for all groups except those between 20–30 and over 60 years of age. Consistent with the report by Villanueva, gender-related variations of serum profiles in healthy individuals were observed only in those 20–30 years old. Since the lung, breast and rectal cancer patients in our study were predominantly 40 or older, age and gender are unlikely to bias the cancer serum profiles. However, age and gender-related variations should be considered when studies involve cancer patients younger than 30 years old.

Other potential bias sources included sample collection, processing and storage, MS detection, and data analysis methods. These sources have been examined in previous studies and remedied in our experiments. Critically important sources of variation include blood collection tubes, clotting times and temperature, and the number of freeze-thaw cycles. Other sources include batch to-batch inconsistency of the magnetic beads, MALDI sample crystallization, and level of laser irradiation [17], [23], [24]. In addition, careful quality control procedures ensured constant peptide extraction and MS detection conditions. The magnetic bead reagent prepared in our lab displayed negligible variation between batches. The high sensitivity and reproducibility of the magnetic-bead-based platform for serum profiling, which was advocated by a recent study from Villanueva et al, were also achieved in our study [14]–[16], [18].

Peptides known to vary naturally with a high incidence (e.g., the 65 peptides observed in over 20% of the healthy cohort in Figure 2) can either be eliminated as diagnostic markers or more rigorously scrutinized for unique variants specific to disease. In contrast, peptides found to vary at a low frequency are of greater interest to biomarker discovery efforts. We observed 3 sets of signatures, each containing 10 discriminating peptides. Each set of 10 peptides was strongly associated with a particular cancer and was not present in the serum profile pattern of the healthy population. We believe the approach and findings described in this report will add to the understanding of serum peptide diversity and have subsequent applications in clinical proteomics.

The identities of both normal serum peptidomes and the peptides comprising the discriminatory ions can potentially lead to insights concerning their sources and relationships to the underlying physiology and pathology. Although advances in MS now make it possible to identify small- to medium-sized peptides in serum using only microliter-volume samples, this technology remains a significant barrier to application to clinical proteomics. The direct identification of peptides in tryptic-digested serum remains difficult because of the complexity of samples. A direct MS/MS identification of the peptides using MALDI TOF/TOF is not possible because of the presence of multiple peptides, even in small mass windows. Although offline nanoLC-MALDI could solve this problem, we think that the best method to identify peptides in complex mixtures is Fourier transform MS (FT-MS), in which the exact mass of the peptide of interest is obtained [25]–[27]. Using FT-MS and enrichment strategies to characterize the low-molecular-weight region of the proteome, we have identified 111 peptides, including 20 peptides with > 20% frequency in healthy sera (Figure 2, Figure 3 and Table 1), 6 discriminatory ions for lung cancer, 4 for rectal cancer, and 8 for breast cancer (Figure 3 and Table 1).

Villanueva et al. reported that sequence identification of 61 signature peptides revealed that they fall into several tight clusters, and most are generated by exopeptidase activities that confer cancer type-specific differences [2]. The serum peptides employed as cancer signatures are not classical markers, but they may serve as an endogenous substrate pool for the real biomarkers, i.e. proteases. Because they are not released by cancers, they likely represent a nonspecific epiphenomenon associated with the presence of cancer [1], [2], [23]. We demonstrated that the majority of the peptides identified in both healthy individuals and cancer patients were clustered into sets of overlapping fragments aligned with ladder-like truncations; most are exopeptidase products of serum resident proteins. How peptide profiling may contribute mechanistically to both cancer-specific and cancer type-specific classifications remains unexplained and may require a great deal of future study to understand. Nonetheless, the differences are statistically significant, holding important information that may have direct clinical utility as surrogate biomarkers in the form of proteome metabolomic products. We believe that proteolytic degradative patterns in the serum peptidome of both cancer patients and healthy controls hold information important for defining cancer-specific peptide profiles that may have direct clinical utility as surrogate biomarkers.

## Supporting Information

Table S1 **Breast cancer patient demographics.**
(DOC)

Table S2 **Rectal cancer patient demographics.**
(DOC)

Table S3 **Non-small-cell lung cancer patient demographics.**
(DOC)

Table S4 **Reproducibility of mass spectra profiled by copper-chelated beads and MALDI-TOF analysis.**
(DOC)
