# Supplementary material for: Serum Peptidome Variations in a Healthy Population: Reference to Identify Cancer-Specific Peptides
Source: PLoS One. 2013 May 8;8(5):e63724. doi: 10.1371/journal.pone.0063724 (PMC3648468; doi:10.1371/journal.pone.0063724)
Supplement: Table S1 — Breast cancer patient demographics. (DOC) [file pone.0063724.s001.doc]

**Table S1. Breast cancer patients demographics.**

| Breast cancer | Whole subjects (84) |
| --- | --- |
| Age  Median(range) | 49.5(30-74) |
| TNF grade |  |
| Grade I | 10(11.9%) |
| Grade II | 47(56.0%) |
| Grade III | 15(17.9%) |
| Grade IV | 12(14.2%) |
| Pathological type |  |
| ductual | 57(67.9%) |
| lobular | 15(17.9%) |
| other | 12(14.2%) |
