# Supplementary material for: Serum Peptidome Variations in a Healthy Population: Reference to Identify Cancer-Specific Peptides
Source: PLoS One. 2013 May 8;8(5):e63724. doi: 10.1371/journal.pone.0063724 (PMC3648468; doi:10.1371/journal.pone.0063724)
Supplement: Table S2 — Rectal cancer patient demographics. (DOC) [file pone.0063724.s002.doc]

**Table S2.** Rectal cancer patients demographics.

| Rectal cancer | Whole subjects (30) |
| --- | --- |
| Age  Median(range) | 56(41-74) |
| Dukes grade |  |
| Grade A | 5(16.7%) |
| Grade B | 9(30.0%) |
| Grade C | 5(16.7%) |
| Grade D | 11(36.6%) |
| Histological type |  |
| adenoid | 26(86.7%) |
| mucous | 3(10.0%) |
| no differentiated | 1(3.3%) |
| Differentiation |  |
| high | 8(26.7%) |
| medium | 13(43.3%) |
| low | 9(30.0%) |
