# Supplementary material for: Serum Peptidome Variations in a Healthy Population: Reference to Identify Cancer-Specific Peptides
Source: PLoS One. 2013 May 8;8(5):e63724. doi: 10.1371/journal.pone.0063724 (PMC3648468; doi:10.1371/journal.pone.0063724)
Supplement: Table S3 — Non-small-cell lung cancer patient demographics. (DOC) [file pone.0063724.s003.doc]

**Table S3.** Non-small-cell lung cancer patients demographics.

| Lung cancer | Whole subjects (70) |
| --- | --- |
| Age  Median(range) | 56(23-79) |
| Pathologic type |  |
| Squamous cell | 48(68.6%) |
| Adeno carcinoma | 22(31.4%) |
| Large cell | 0(0%) |
| Stage grouping |  |
| Stage 0 | 3(4.3%) |
| Stage IA | 6(8.6%) |
| Stage IB | 13(18.6%) |
| Stage IIA | 4(5.7%) |
| Stage IIB | 8(11.4%) |
| Stage IIIA | 13(18.6%) |
| Stage IIIB | 12(47.1%) |
| Stage IV | 11(15.7%) |
