# Supplementary material for: Serum Peptidome Variations in a Healthy Population: Reference to Identify Cancer-Specific Peptides
Source: PLoS One. 2013 May 8;8(5):e63724. doi: 10.1371/journal.pone.0063724 (PMC3648468; doi:10.1371/journal.pone.0063724)
Supplement: Table S4 — Reproducibility of mass spectra profiled by copper-chelated beads and MALDI-TOF analysis. (DOC) [file pone.0063724.s004.doc]

**Table S4.** Reproducibility of mass spectra profiled by copper-chelated beads and MALDI-TOF analysis.

| Peak (m/z) | Within-day | | |  | Between-day | | |
| --- | --- | --- | --- | --- | --- | --- | --- |
|  | Intensity^a^ | SD | ICV,% |  | Intensity^a^ | SD | ICV,% |
| 1933.52 | 0.1075 | 0.0057 | 5.30 |  | 0.1078 | 0.0059 | 5.47 |
| 2012.39 | 0.0183 | 0.0016 | 8.74 |  | 0.0182 | 0.0019 | 10.44 |
| 2545.69 | 0.0167 | 0.0013 | 7.78 |  | 0.0163 | 0.0018 | 11.04 |
| 2863.06 | 0.0102 | 0.0011 | 10.78 |  | 0.0098 | 0.0010 | 10.20 |
| 3158.59 | 0.0265 | 0.0028 | 10.57 |  | 0.0271 | 0.0026 | 9.59 |
| 3254.85 | 0.0138 | 0.0015 | 10.87 |  | 0.0138 | 0.0014 | 10.14 |
| 3212.05 | 0.0171 | 0.0019 | 11.11 |  | 0.0176 | 0.0020 | 11.36 |
| 4269.96 | 0.0462 | 0.0045 | 9.74 |  | 0.0459 | 0.0043 | 9.37 |
| 6626.75 | 0.1613 | 0.0092 | 5.70 |  | 0.1617 | 0.0099 | 6.12 |
| 7740.77 | 0.0091 | 0.0011 | 12.09 |  | 0.0103 | 0.0014 | 13.59 |

ICV, individual CV of each peak. SD, Standard deviation of each peak. Intensity^a^, the average intensity of peak in 6 spectra.
